# Supplementary material for: Dynamical latent state computation in the male macaque posterior parietal cortex
Source: Nat Commun. 2023 Apr 1;14:1832. doi: 10.1038/s41467-023-37400-4 (PMC10067966; doi:10.1038/s41467-023-37400-4)
Supplement: Supplementary file 3 — Reporting Summary [file 41467_2023_37400_MOESM3_ESM.pdf]

# nature portfolio

Corresponding author(s): Kaushik Lakshminarasimhan

Last updated by author(s): Dec 12, 2022

# Reporting Summary

Nature Portfolio wishes to improve the reproducibility of the work that we publish. This form provides structure for consistency and transparency in reporting. For further information on Nature Portfolio policies, see our Editorial Policies and the Editorial Policy Checklist.

## Statistics

For all statistical analyses, confirm that the following items are present in the figure legend, table legend, main text, or Methods section.

| n/a | Confirmed | |
|---|---|---|
| ☐ | ☒ | The exact sample size (*n*) for each experimental group/condition, given as a discrete number and unit of measurement |
| ☐ | ☒ | A statement on whether measurements were taken from distinct samples or whether the same sample was measured repeatedly |
| ☐ | ☒ | The statistical test(s) used AND whether they are one- or two-sided<br>*Only common tests should be described solely by name; describe more complex techniques in the Methods section.* |
| ☐ | ☒ | A description of all covariates tested |
| ☒ | ☐ | A description of any assumptions or corrections, such as tests of normality and adjustment for multiple comparisons |
| ☐ | ☒ | A full description of the statistical parameters including central tendency (e.g. means) or other basic estimates (e.g. regression coefficient) AND variation (e.g. standard deviation) or associated estimates of uncertainty (e.g. confidence intervals) |
| ☐ | ☒ | For null hypothesis testing, the test statistic (e.g. *F*, *t*, *r*) with confidence intervals, effect sizes, degrees of freedom and *P* value noted<br>*Give P values as exact values whenever suitable.* |
| ☒ | ☐ | For Bayesian analysis, information on the choice of priors and Markov chain Monte Carlo settings |
| ☒ | ☐ | For hierarchical and complex designs, identification of the appropriate level for tests and full reporting of outcomes |
| ☐ | ☒ | Estimates of effect sizes (e.g. Cohen's *d*, Pearson's *r*), indicating how they were calculated |

*Our web collection on statistics for biologists contains articles on many of the points above.*

## Software and code

Policy information about availability of computer code

| | |
|---|---|
| Data collection | Cereplex Direct Software Suite from Blackrock Microsystems: version 6.x<br>Spike2 from Cambridge Electronics Devices: version 6.18 |
| Data analysis | DeepLabCut: https://github.com/DeepLabCut/DeepLabCut/releases/tag/1.11<br>Custom MATLAB code: https://github.com/kaushik-l/firefly-monkey \| https://github.com/kaushik-l/neuroGAM |

For manuscripts utilizing custom algorithms or software that are central to the research but not yet described in published literature, software must be made available to editors and reviewers. We strongly encourage code deposition in a community repository (e.g. GitHub). See the Nature Portfolio guidelines for submitting code & software for further information.

## Data

Policy information about availability of data

All manuscripts must include a data availability statement. This statement should provide the following information, where applicable:

- Accession codes, unique identifiers, or web links for publicly available datasets
- A description of any restrictions on data availability
- For clinical datasets or third party data, please ensure that the statement adheres to our policy

All pre-processed data are available on g-node
Raw data files are quite large (~50GB/experimental session) and stored in a local database but can be shared upon reasonable request

## Human research participants

Policy information about studies involving human research participants and Sex and Gender in Research.

| | |
|---|---|
| Reporting on sex and gender | N/A |
| Population characteristics | N/A |
| Recruitment | N/A |
| Ethics oversight | N/A |

Note that full information on the approval of the study protocol must also be provided in the manuscript.

# Field-specific reporting

Please select the one below that is the best fit for your research. If you are not sure, read the appropriate sections before making your selection.

☒ Life sciences ☐ Behavioural & social sciences ☐ Ecological, evolutionary & environmental sciences

For a reference copy of the document with all sections, see nature.com/documents/nr-reporting-summary-flat.pdf

# Life sciences study design

All studies must disclose on these points even when the disclosure is negative.

| | |
|---|---|
| Sample size | The number of monkeys (n=3) is greater than the standard sample size in the field of primate neuroscience (typically n=2). The number of trials for behavioral analyses (n>100,000) is much greater than the standard sample size in systems neuroscience (n< 10,000) used to calculate ROC curves. The number of neurons (n=244) is standard in studies that perform simultaneous recordings for the purpose of neural decoding. The minimum number of neurons required has been argued to be not much greater than the dimensionality of neural activity, and simple neuroscience tasks tend to observe a dimensionality of less than 5 (Gao et al. 2017; https://www.biorxiv.org/content/10.1101/214262v2). Although the task used here is naturalistic and more complex, we did not expect the dimensionality to be orders of magnitude greater than standard tasks and therefore used Utah arrays which yielded about 80 neurons per session. As expected, the dimensionality of neural activity (quantified using participation ratio) was found to be less than 10 and the decoders are demonstrably robust to subsampling, thus validating our assumptions |
| Data exclusions | Trials in which the monkeys did not perform the navigation task were excluded. This is similar to excluding trials in which the monkey does not fixate in more traditional experiments. For the main results, we chose experimental sessions with the highest yield |
| Replication | Data collected from the third monkey validated all experimental findings from analyzing data from the first two monkeys |
| Randomization | Trials were randomized such that the target locations were unpredictable. When using experimental manipulations, manipulated trials were randomly interleaved except for one experimental manipulation which was performed in blocks (gain manipulation) |
| Blinding | The study did not divide monkeys into groups, so blinding was not necessary |

# Reporting for specific materials, systems and methods

We require information from authors about some types of materials, experimental systems and methods used in many studies. Here, indicate whether each material, system or method listed is relevant to your study. If you are not sure if a list item applies to your research, read the appropriate section before selecting a response.

### Materials & experimental systems

| n/a | Involved in the study | |
|---|---|---|
| ☒ | ☐ | Antibodies |
| ☒ | ☐ | Eukaryotic cell lines |
| ☒ | ☐ | Palaeontology and archaeology |
| ☐ | ☒ | Animals and other organisms |
| ☒ | ☐ | Clinical data |
| ☒ | ☐ | Dual use research of concern |

### Methods

| n/a | Involved in the study | |
|---|---|---|
| ☒ | ☐ | ChIP-seq |
| ☒ | ☐ | Flow cytometry |
| ☒ | ☐ | MRI-based neuroimaging |

## Animals and other research organisms

Policy information about studies involving animals; ARRIVE guidelines recommended for reporting animal research, and Sex and Gender in Research

| | |
|---|---|
| Laboratory animals | Rhesus macaques; all male; 7-8 years old |
| Wild animals | No wild animals were used in this study |
| Reporting on sex | All animals were male as reported in the methods section |
| Field-collected samples | No field-collected samples were used in this study |
| Ethics oversight | All surgeries and experimental procedures were approved by the institutional review board at Baylor College of Medicine, and were in accordance with the National Institutes of Health guidelines |

Note that full information on the approval of the study protocol must also be provided in the manuscript.
